# Characteristics in gut microbiome is associated with chemotherapy-induced pneumonia in pediatric acute lymphoblastic leukemia

**DOI:** 10.1186/s12885-021-08917-y

**Published:** 2021-11-08

**Authors:** Xiaoming Liu, Yao Zou, Yingchi Zhang, Lipeng Liu, Yongjuan Duan, Aoli Zhang, Xiaoyan Zhang, Ranran Zhang, Beibei Zhao, Xiaolan Li, Tong Wei, Hongrui He, Yu Gan, Kejian Wang, Xiaofan Zhu

**Affiliations:** 1 State Key Laboratory of Experimental Hematology, National Clinical Research Center for Blood Diseases, Division of Pediatric Blood Diseases Center, Institute of Hematology & Blood Diseases Hospital, Chinese Academy of Medical Sciences & Peking Union Medical College, Tianjin, 300020 China; 2 College of Biological Science and Engineering, Fuzhou University, Fuzhou, China; 3 Lin He’s Academician Workstation of New Medicine and Clinical Translation at The Third Affiliated Hospital, Guangzhou Medical University, Guangzhou, China; 4 The Third Affiliated Hospital of Shandong First Medical University (Affiliated Hospital of Shandong Academy of Medical Sciences), Jinan, 250031 China; 5 Gastroenterology Research Institute and Clinical Center, Shandong First Medical University (Shandong Academy of Medical Sciences), Jinan, 250031 China

**Keywords:** Pediatric acute lymphoblastic leukemia, Chemotherapy-induced pneumonia, Gut microbiome, 16S rRNA quantitative microarray

## Abstract

**Background:**

Children with acute lymphoblastic leukemia (ALL) undergoing chemotherapy experience a relatively high risk of infection. And the disturbance of gut microbiota is generally believed to impair intestinal barrier function and may induce bacterial infections and inflammation. The study aimed to investigate the alterations in the gut microbiota and assess its relationship with chemotherapy-induced pneumonia in pediatric ALL patients.

**Methods:**

We conducted a case–control study with 14 cases affected by pneumonia and 44 unaffected subjects and characterized the physiological parameters and gut microbiota by microarray-based technique.

**Results:**

There were significant differences in α- and β-diversity in the affected group compared with the control group. At species level, the LEfSe analysis revealed that *Enterococcus malodoratus*, *Ochrobactrum anthropi* and *Actinomyces cardiffensis* were significantly abundant in the affected subjects. A receiver operating characteristic (ROC) curve yielded the area under the curve (AUC) of 0.773 for classification between the two groups. In addition, the Kyoto Encyclopedia of Genes and Genomes (KEGG) pathways involved in the bacterial secretion system were more enriched in the affected group than in the control group.

**Conclusions:**

Gut microbiota alteration was associated with chemotherapy-induced pneumonia in pediatric ALL patients, which provided a new perspective on the personalized clinical care of pediatric ALL.

**Supplementary Information:**

The online version contains supplementary material available at 10.1186/s12885-021-08917-y.

## Background

Acute lymphoblastic leukemia (ALL) is a malignant neoplastic disease with an uncontrollable proliferation of immature leukocytes and primarily inhibits normal hematopoietic function [1]. At present, chemotherapy is one of the dominant treatment methods for ALL [2]. However, patients in the chemotherapy period often suffer from low immune function and subsequent infection. More than 80% of patients may have fever during agranulocytosis [3]. Pneumonia is one of the most common infectious complications, which develops in 13 to 31% of ALL patients and has fatality rates of 25 to 45% [4].

Recently, increasing attention has been drawn to the research of gut microbiota due to its role in human health [5, 6]. Accumulating evidence have shown that microbial dysbiosis is related to a variety of respiratory diseases such as pneumonia, chronic obstructive pulmonary disease, and respiratory syndrome [7, 8]. It is also widely known that the disruption of the gut microbiome may lead to the development of many different diseases, including metabolic syndrome [9], atherosclerosis [10, 11], and frailty [12]. The alterations in the diversity and composition of gut microbiota also play a major role in predicting infection in ALL children and adults by chemotherapy. Hakim et al. reported that in the ALL child undergoing chemotherapy, the decreasing abundance of certain bacteria predicted infections in subsequent phases [13]. Chua et al. found that gut microbiota in children with ALL differed from that in healthy controls, which is linked with chemotherapy-induced infection [14]. Figuring out the interactions between gut microbial communities and pneumonia status of ALL survivors may be a potential strategy for personalized health care. Hence, we utilized a new microarray-based technique to assess the correlation between the gut microbiome and chemotherapy-induced pneumonia in pediatric ALL patients.

Here, we investigated the association between gut microbiota and the pediatric ALL patients with chemotherapy-induced pneumonia, so as to understand the role played by specific bacterial taxa.

## Methods

### Study design and participants

From 1 November 2018 to 31 March 2019, 58 patients with diagnosis of ALL were recruited into the study. Patients were 3–15 years old at the time of diagnosis. A written informed consent was obtained from patients or legal guardians. All patients were diagnosed based on morphologic, cytochemical and immunophenotypic criteria, and met the criteria for inclusion and classification of the CCCG (Chinese Children’s Cancer Group) ALL2015 protocol [15]. Children who have been treated with antibiotics or intestinal probiotics within 14 days before feces collection are not in the scope of this study.

All children received induction therapy according to their risk in CCCGALL2015. Patients’ demographic data, leukemia information, compliance to chemotherapy and adverse events including pneumonia were regular reporting events. Any 3 of the following 4 items in children with ALL during or after chemotherapy were defined as chemotherapy-induced pneumonia: 1) fever as an axillary temperature > 38.0 °C persisting for > 1 h; 2) Accompanied by symptoms such as cough, sputum, chest tightness, and suffocation; 3) X-ray or CT of the lungs suggested pulmonary infection with direct or indirect evidence of bacterial or viral or fungal infection; 4) Fingertip blood oxygen saturation was lower than 95%; 5) Neutrophils less than 0.5 × 10^9^/L [16].

According to CCCGALL2015 protocol [17, 18], induction chemotherapy of “VDLD (vincristine + daunorubicin + PEG-Asp + Prednisone) + CAM (cyclophosphamide + cytarabine + mercaptopurine)” was 36–40 days in total, and the recovery period after chemotherapy was about 10–20 days. Therefore, the scope of this study was defined as the patient’s situation from the start of induction chemotherapy to 60 days. Pneumonia occurring before or 60+ days after induction chemotherapy was not in the scope of this study. And all the children in this study had not used antibiotics before taking stool samples.

According to the imaging characteristics of pneumonia, pneumonia is divided into: lobar pneumonia, lobular pneumonia, and interstitial pneumonia. According to the pathogenic examination and related infection indicators (C-reactive protein (CRP), procalcitonin, endotoxin, 1–3-β-D-glucan test (G Test), galactomannan test (GM Test), aspergillus antibody, candida antibody, mycoplasma pneumoniae antibody, etc.), pneumonia was divided into bacterial pneumonia, fungal pneumonia, viral pneumonia, and multifactorial pneumonia. All positive indicators were tested once a week until they turned negative. Due to insufficient spitting ability in children, no valuable sputum culture results have been obtained. Due to the general bleeding tendency during induction therapy in children with leukemia, the etiological examination of bronchial washing was not available.

This study protocol was approved by the Research Ethics Committee, Institute of Hematology & Blood Diseases Hospital, Chinese Academy of Medical Sciences & Peking Union Medical College, State Key Laboratory of Experimental Hematology, National Clinical Research Center for Blood Diseases, Tianjin, China (No. KT2018099-EC-1).

### Sample collection

Stool specimens were obtained when the disease was diagnosed but no any treatment was started. All fecal specimens were kept in the hospital under the guidance of nurses. To protect the integrity of 16S rRNA, according to the manufacturer’s instructions (Halgen, Guangdong, China), the fresh feces collected were directly placed in a preservation solution containing 1% SDS, 20 mmol EDTA and Proteinase K, and immediately stored at − 80 °C for testing.

### DNA extraction

We used Halgen Stool Sampler to take or weight 150–250 mg fecal sample and transfer to the Lysis Buffer tube. In brief, the DNA extraction was composed of chemical and mechanical lysis follow the kit instructions. The purified DNA was stored at − 20 °C until use.

### 16S microarray assay

The 16S microarray assay was conducted following our previously published protocols [19]. All variable regions of bacterial 16S rRNA were selected as probes. The length of the probe was about 40 bp. The DNA of the test sample and the labeled reference pool were both hybridized mixtures to accurately identify the spot and quantify the signal. Heat the Cy3- and Cy5-labeled samples and hybridization buffer to 100 °C for 5 min, cooled for 5 min, and hybridized at 37 °C for 3.5 h. Washed the slides at 63 °C for 15 min, and scanned the slides with a dual-channel scanner. In brief, bacterial DNA was extracted from the stool samples by using a stool DNA extraction kit (Halgen, Guangdong, China) and amplified for 16S rRNA gene regions V1-V9 in PCR. The PCR products were directly labeled for hybridization with species-specific probes on the microarray. The relative abundance of each bacterial species was determined by the Cy5 / Cy3 ratios of each probe (with proprietary software provided by Halgen, Guangdong, China).

### Data analysis

Wilcoxon rank-sum test was used to measure the disparities in α-diversity between groups. Principal coordinate analysis (PCoA) was performed using Quantitative Insights Into Microbial Ecology (QIIME) modules and visualized with R packages (version 3.5.2). Linear Discriminant Analysis (LDA) Effect Size (LEfSe) analysis was carried out to analyze the difference between groups of bacterial species. For each species, the *p*-value was calculated through the Kruskal-Wallis test and the Wilcoxon test. Also using R, unmonitored random forest clustering and the receiver operating characteristic curve (ROC curve) were performed with proportional hazard statistics. Cross-validation was conducted by leave-one-out check in random forest clustering to reduce the effect of overfitting. Phylogenetic Investigation of Communities by Reconstruction of Unobserved States (PICRUSt) [20] was employed to predict the functions of differentially abundant tax with reference to the Kyoto Encyclopedia of Genes and Genomes (KEGG) pathways.

## Results

### Characteristics of study subjects

The demographic characteristics of affected and control groups were summarized in Table 1. Except for white blood cell (*P* = 0.001) and blasts in bone marrow (*P* = 0.017), there was no significant difference in other clinical parameters between the two groups (Table 1).

**Table 1:** Comparison of baseline clinical characteristics

	Affected group	Control group	*P*-value
	(*n* = 14)	(*n* = 44)	
Age [year, median (range)]	8.4 (3.0–15.0)	6.8 (3.0–14.0)	0.433
Male (n, %)	10 (71.4)	24 (54.5)	0.356
WBC [×10^9^/L, median (range)]	51.8 (1.6–344.4)	13.2 (0.8–189.6)	**0.001**
Hemoglobin [g/L, median (range)]	86.9 (58.0–135.0)	86.0 (42.0–136.0)	0.353
Platelet [×10^9^/L, median (range)]	58.6 (10.0–239.0)	97.3 (9.0–321.0)	0.061
blasts in BM (%)	85.6 (63.5–96.5)	71.8 (20.0–94.7)	**0.017**
CNS involvement (CNS2/3) (n, %)	2 (14.3)	3 (6.8)	0.585
Hepatosplenomegaly (n, %)	4 (28.6)	21 (47.7)	0.235
Swollen lymph nodes (n, %)	4 (28.6)	15 (34.1)	0.468
Immunophenotype			0.673
B-ALL	11 (78.6)	38 (86.4)	
T-ALL	3 (21.4)	6 (13.6)	
Risk Group			0.338
low-risk (LR)	7 (50.0)	30 (68.2)	
intermediate-risk (IR)	7 (50.0)	14 (31.8)	
Birth order			0.802
first born (n, %)	10 (71.4)	29 (65.9)	
second child (n, %)	3 (21.4)	13 (29.5)	
third and upper (n, %)	1 (7.1)	2 (4.5)	
Delivery			0.568
spontaneous delivery	8 (57.1)	26 (59.1)	
caesarean section	6 (42.9)	18 (40.9)	
Birth weight			0.513
less than or equal to 3 kg (n, %)	3 (21.4)	15 (34.1)	
more than 3 kg (n, %)	11 (78.6)	29 (65.9)	
Feeding patterns			0.711
breastfeeding (n, %)	12 (85.7)	34 (77.3)	
non-breastfeeding (n, %)	2 (14.3)	10 (22.7)	
Diet			0.676
not picky eaters (n, %)	10 (71.4)	34 (77.3)	
meat-based (n, %)	3 (21.4)	9 (20.5)	
vegetarian-based (n, %)	1 (7.1)	1 (2.3)	
Constipation (n, %)	3 (21.4)	6 (13.6)	0.673
BMI [kg/m2, median (range)]	31.0 (25.4–37.5)	26.8 (21.5–38.8)	0.152

*WBC* White blood cell, *CNS* Central nervous system, *BM* bone marrow, *BMI* Body Mass Index

Among 58 ALL patients, 14 cases were with pneumonia within the study period and 44 controls were without pneumonia. Of the 14 children with pneumonia, 8 cases occurred during induction chemotherapy, and 6 cases occurred after induction chemotherapy; 4 cases of lobar pneumonia, 4 cases of lobular pneumonia, and 6 cases of interstitial pneumonia; 2 cases of bacterial pneumonia, 1 case of viral pneumonia, 5 cases of fungal pneumonia, and 6 cases of multifactorial pneumonia. The specific conditions of 14 cases of pneumonia in children were shown in Supplementary Table 1.

### Gut microbiome composition of patients

**Fig. 1:**
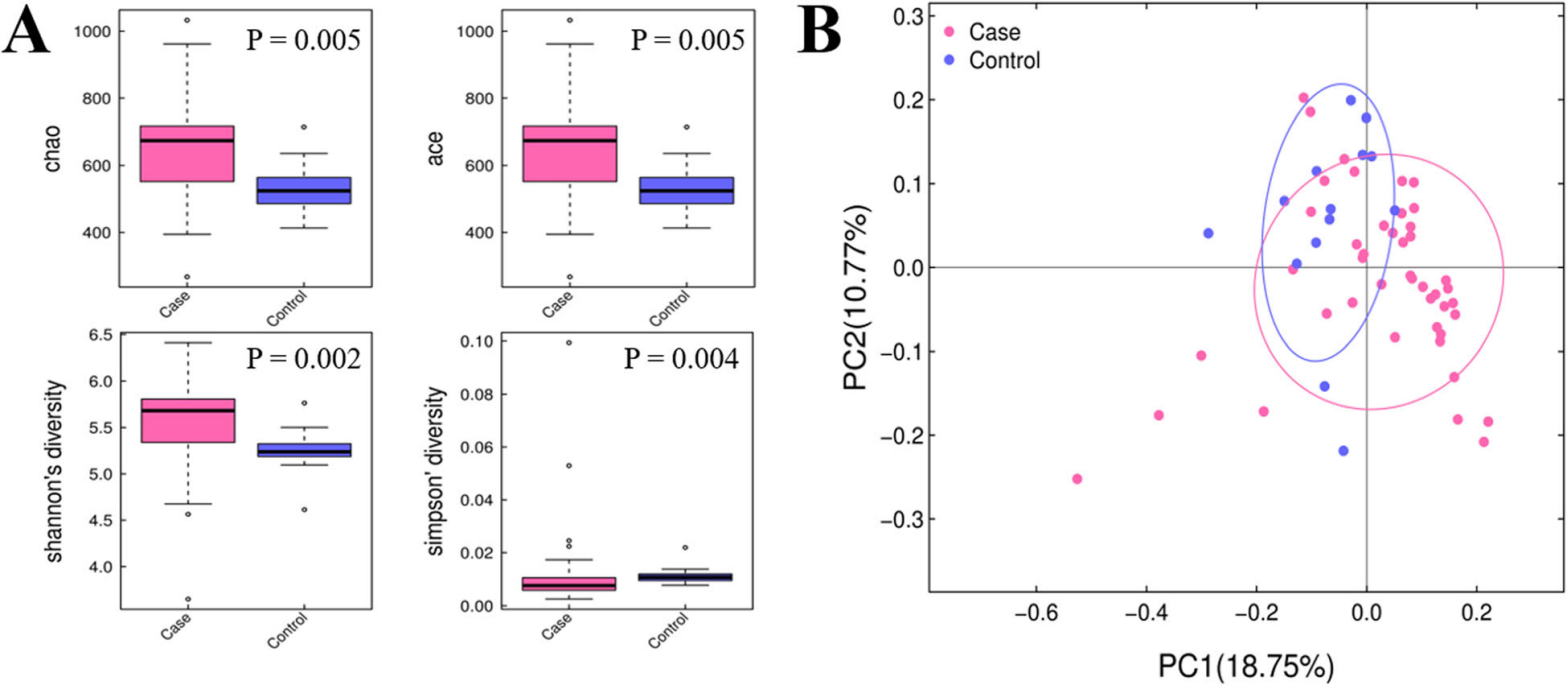
The α- and β-diversity of gut microbiota of all samples. **A** Boxplots of Chao, Ace, Simpson, and Shannon indexes for comparison of α- diversity. **B** Comparison of β-diversity between the microbiota of cases and controls. PCoA plot demonstrates the distinction between affected and control groups

To characterize the bacterial richness and diversity, Chao, Ace, Simpson, and Shannon indexes were calculated to estimate α-diversity. We found a significant difference in various indexes between the two groups (Fig. 1A), showing a higher diversity in pneumonia cases than in unaffected subjects. In addition, Bray-Curtis distance was used to measure β-diversity. As shown in Fig. 1B, the subjects of the two groups had a clear separation, which revealed the difference in bacterial communities between affected and unaffected groups (PERMANOVA, permutational multivariate analysis of variance, *P*-value = 0.001).

### Differential microbiota compositions

**Fig. 2:**
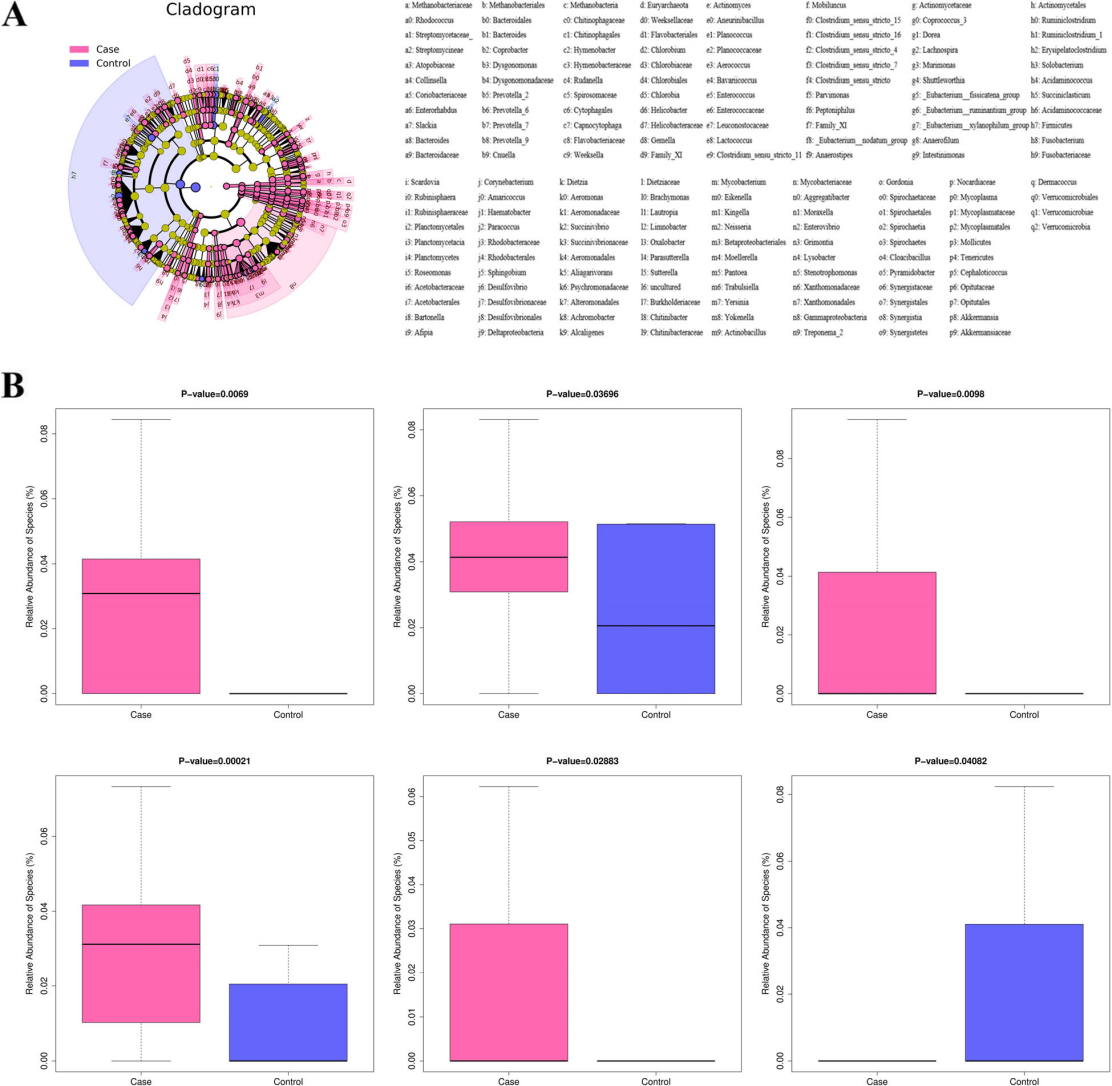
Differentially abundant taxa associated with chemotherapy-induced pneumonia. **A** Cladogram representing linear discriminant analysis effect size results. **B** Relative abundance of specific microbiota taxa differentially enriched in the case or control group

We used the LEfSe tool to identify taxon with differential abundance between two groups (Fig. 2A). Significant variations in the bacterial communities were observed at the species level. And a series of species (including *Enterococcus malodoratus*, *Ochrobactrum anthropi*, and *Actinomyces cardiffensis*) were relatively enriched in the affected group than those in the control group, while the abundances of *Bacillus altitudinis*, *Afipia birgiae*, and *Bifidobacterium tsurumiense* were significantly higher in controls than in cases (Fig. 2B). The results of LEfSe analysis suggested that the gut microbial composition was differential.

**Fig. 3:**
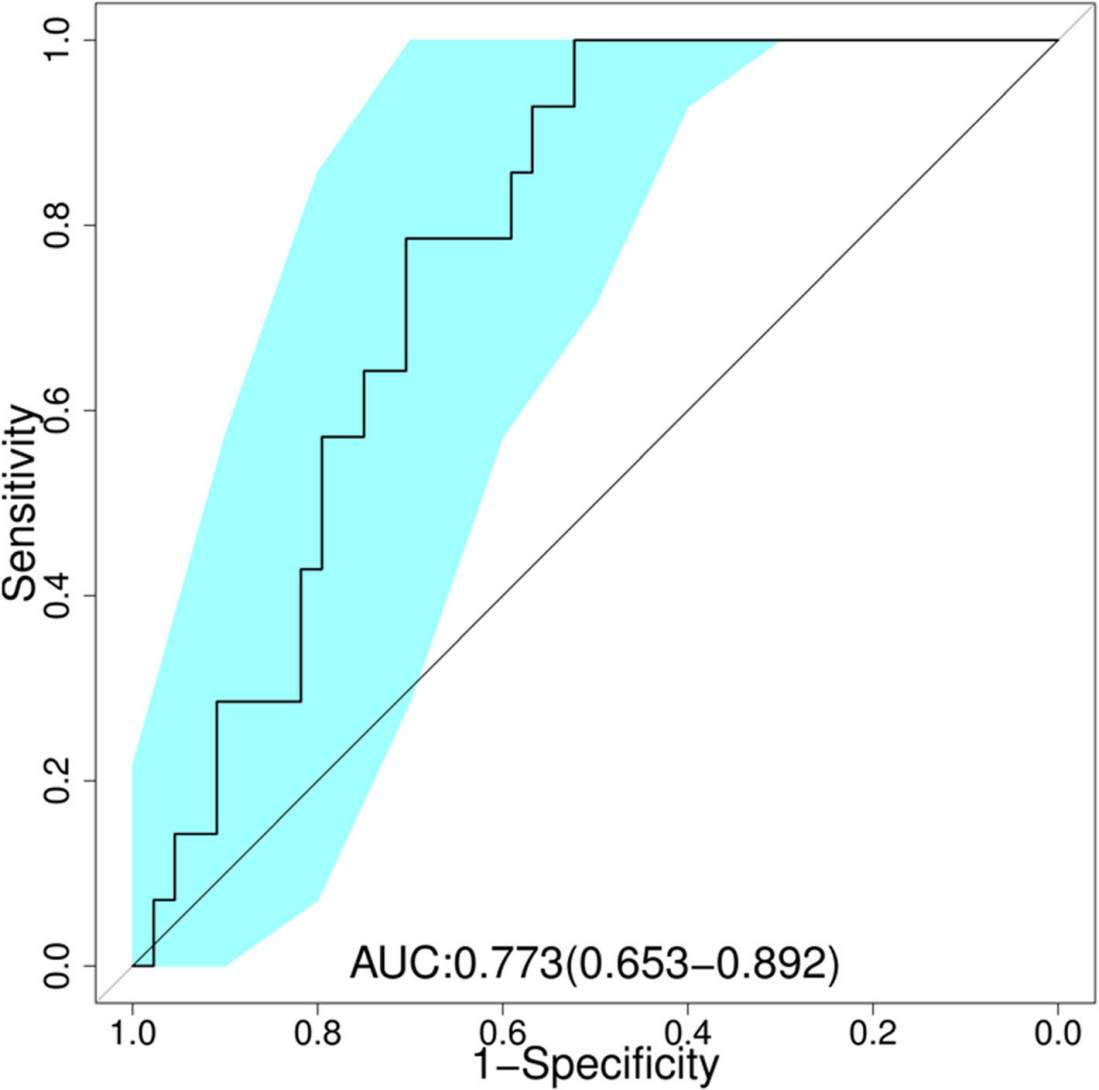
Receiver operating characteristic (ROC) curves with species

To measure whether the relative abundance of species could distinguish samples of two groups, we performed classification using the random forests algorithm. Receiver operating characteristic (ROC) curve was applied to evaluate the performance of prediction model (Fig. 3). The value of area under curve (AUC) was 0.773, suggesting an effective performance for subject discrimination and prediction.

### Functional differences in gut microbiome among ALL patients

**Fig. 4:**
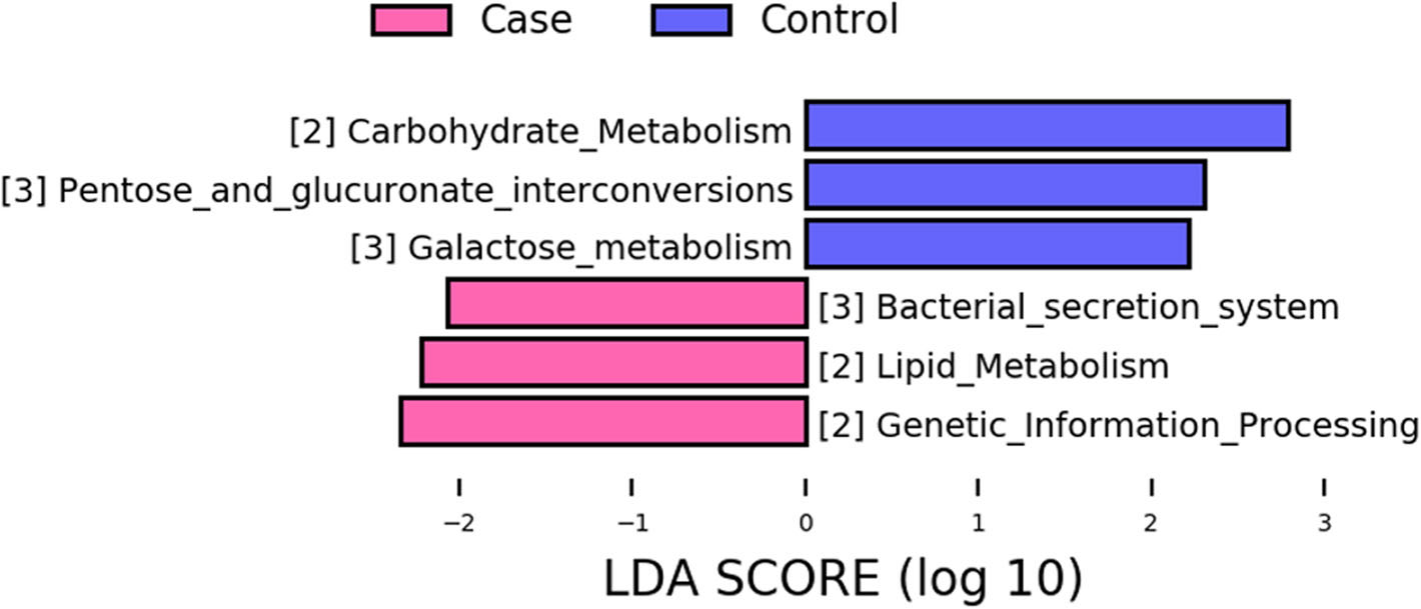
Metabolic pathway differed between case and control groups

Subsequently, we further assessed functional annotations of pneumonia-associated microbial taxa (Fig. 4). Kyoto Encyclopedia of Genes and Genomes (KEGG) pathway analysis suggested that the pneumonia-associated taxa were enriched in several pathways such as lipid metabolism, genetic information processing, and bacterial secretion system. Among them, the bacterial secretion system is worth noting due to its association with the host’s immune system [21]. These results revealed the differences in the potential function of microbiome between the two groups.

## Discussion

In this study, we demonstrated the significant differences in gut microbial composition and diversity between affected and control groups. And differential abundance of taxa at species level was shown in LEfSe analysis. Besides, KEGG indicated the changes in metabolic pathways between the two groups.

The α-diversity indices, including Chao, Ace, Simpson, and Shannon indexes, displayed a higher bacterial richness and diversity, which was in contrast to previous studies that reported reduced diversity in cancer patients [22]. It has raised a possibility that cancer-related treatment such as chemotherapy disrupts intestinal microbiota homeostasis of ALL patients, facilitating the overgrowth of harmful bacteria [23].

Regarding β-diversity, Bray-Curtis analysis was used to compare the similarity and dissimilarity of gut microbial communities, which revealed a clear separation in the affected and control groups. The distinctive features of the two groups might mirror the association between the gut microbiota and chemotherapy-induced pneumonia.

We then performed LEfSe analysis to identify differential taxon and screen key species. The abundance of *Enterococcus malodortheatus*, *Actinomyces cardiffensis*, and *Ochrobactrum anthropic* were increased in the affected group as compared to the control group. Previous studies have reported that Enterococcus spp. is associated with respiratory tract infections [24], and *Actinomyces cardiffensis* is a definite risk factor of pneumonia [25]. *Ochrobactrum anthropic*, a type of Gram-positive bacteria, is associated with pyogenic infections [26]. Naik et al. suggested an increased relative abundance of *Ochrobactrum anthropic* in serious infections including pneumonia [27].

It is well documented that altered gut microbiota may provoke distinctive inflammatory responses. A study by St Jude Children’s Research Hospital showed that after chemotherapy in children with acute lymphoblastic leukemia, the relative abundance of certain bacterial groups (such as Bacteroides) decreased significantly, while the relative abundance of other groups (such as Clostridia and Streptococcus) The relative abundance increases. The baseline gut microbiome characterized by Proteobacteria predicts febrile neutropenia [13]. A recent study discussed the influence of gut microbiota composition on pulmonary tuberculosis patients, which demonstrated that human gut and lung occurs cross-linking through a gut-lung axis [28]. Several studies have emphasized the effect of gut microbiota on lung immunity and underlying links between the specific gut microbiota with lung immunity or respiratory diseases [29, 30].

In addition to the results we presented above, further investigation into the relationship between bacterial functions and chemotherapy-induced pneumonia was also examined. According to the KEGG pathway analysis, we observed an enrichment of differentially abundant taxa in the bacterial secretion system pathway. Bacterial secretion system its responsible for transporting proteins and nucleic acids across cell membranes and linkage to virulence and interaction with the host’s immune system, which is reported to cause extensive pulmonary infections [31]. Therefore, we speculated that certain highly abundant bacteria in the affected group might play important role in the pathogenesis of chemotherapy-induced pneumonia via affecting bacterial secretion system.

One outstanding technical advantage is the application of microarray instead of sequencing to quantify bacterial 16S rRNA, which is different from traditional methods of intestinal microbiota research. Due to the limitation of the length of sequencing reads, traditional 16S rRNA sequencing can only cover a relatively short area, and the level of abundance data is limited to genera. The 16S rRNA quantitative microarray quantifies various bacterial taxa at the species level. In particular, our previous studies [19] have shown that qPCR experiments can effectively verify the differential abundance identified by microarrays. Microarray technology allows us to conduct a more in-depth analysis of the intestinal microbiota, which helps to thoroughly explain the relationship between intestinal microflora imbalance and chemotherapy-related pneumonia in children with ALL.

However, several limitations of this study should also be noticed. Firstly, despite strict inclusion criteria, we cannot completely rule out the age, gender, diet, and BMI that might contribute to dysbiosis. Thus, detailed dietary information is required in our subsequent study. Secondly, all participants were recruited from the same region, and the sample size was relatively small. Therefore, we need to consider a larger cohort and select multi-ethnic patients for powerfully longitudinal studies to further support our findings. Third, due to limited clinical testing methods, direct evidence of the etiology of pneumonia is lacking. Bronchial washing under safety guarantees to look for pathogenic evidence or further needs. In addition, neutropenic fever, oral ulcers, infectious diarrhea, bacteremia, and other complications are also common complications in the treatment of leukemia. We are actively studying the relationship between gut microbiota and these complications. Related articles are being written.

## Conclusion

We found that altered gut microbiota was associated with chemotherapy-induced pneumonia in pediatric ALL patients. Our study provided novel insights into potential links between the gut microbiome and ALL outcome, which contributes to the preventive and therapeutic interventions of ALL patients with pneumonia.

## Supplementary Information

**Additional file 1.**

## Data Availability

The datasets used and/or analysed during the current study are available from the corresponding author on reasonable request.

## Abbreviations

ALL: Acute lymphoblastic leukemia
AUC: Area under the curve
BM: Bone marrow
BMI: Body Mass Index
CCCG: Chinese Children’s Cancer Group
CNS: Central nervous system
KEGG: Kyoto Encyclopedia of Genes and Genomes
LDA: Linear Discriminant Analysis
LEfSe: Effect Size
PCoA: Principal coordinate analysis
PERMANOVA: Permutational multivariate analysis of variance
QIIME: Quantitative Insights Into Microbial Ecology
ROC: Receiver operating characteristic
WBC: White blood cell
