# Supplementary material for: Characteristics in gut microbiome is associated with chemotherapy-induced pneumonia in pediatric acute lymphoblastic leukemia
Source: BMC Cancer. 2021 Nov 8;21:1190. doi: 10.1186/s12885-021-08917-y (PMC8577014; doi:10.1186/s12885-021-08917-y)
Supplement: Supplementary file 1 — Additional file 1. [file 12885_2021_8917_MOESM1_ESM.docx]

Supplementary Table 1: Specific conditions related to pneumonia in 14 children with pneumonia

| **PN** | **SP** | **MT (days)** | **Type 1** | **Type 2** | **Procalcitonin** | **Endotoxin** | **CRP** | **G-T** | **GM-T** | **AA** | **CA** | **MPA** | **T (℃)** | **DF (days)** | **DA (day)** | **Outcome** |
| --- | --- | --- | --- | --- | --- | --- | --- | --- | --- | --- | --- | --- | --- | --- | --- | --- |
| **1** | DT | 20 | Lobular P | multifactorial | High | High | High | Negative | Negative | Negative | Negative | Negative | 39.0 | 4 | 40 | Survival |
| **2** | AT | 50 | Interstitial P | multifactorial | High | High | High | Negative | Negative | Negative | Negative | Negative | 39.2 | 1 | 24 | Survival |
| **3** | DT | 19 | Interstitial P | viral | Normal | Normal | High | Negative | Negative | Negative | Negative | Negative | 39.5 | 4 | 35 | Survival |
| **4** | AT | 41 | Lobular P | fungal | High | Normal | Normal | Negative | Negative | Positive | Positive | Negative | 38.8 | 3 | 48 | Survival |
| **5** | AT | 44 | Lobar P | multifactorial | High | Normal | Normal | Negative | Negative | Negative | Negative | Negative | 40.0 | 9 | 26 | Survival |
| **6** | DT | 11 | Interstitial P | fungal | High | High | Normal | Negative | Negative | Positive | Positive | Negative | 38.7 | 1 | 45 | Survival |
| **7** | AT | 49 | Interstitial P | fungal | Normal | High | High | Negative | Negative | Positive | Positive | Negative | 39.9 | 6 | 21 | Survival |
| **8** | DT | 20 | Interstitial P | fungal | Normal | Normal | Normal | Positive | Positive | Negative | Negative | Negative | 39.8 | 7 | 38 | Survival |
| **9** | DT | 30 | Lobular P | bacterial | Normal | Normal | High | Negative | Negative | Negative | Negative | Negative | 39.5 | 1 | 36 | Survival |
| **10** | AT | 45 | Lobar P | bacterial | High | Normal | Normal | Negative | Negative | Negative | Negative | Negative | 40.0 | 1 | 30 | Die |
| **11** | AT | 43 | Lobar P | multifactorial | High | High | Normal | Negative | Negative | Positive | Positive | Negative | 40.0 | 22 | 20 | Survival |
| **12** | DT | 30 | Lobar P | multifactorial | High | Normal | High | Negative | Negative | Negative | Negative | Negative | 39.0 | 11 | 41 | Survival |
| **13** | DT | 36 | Interstitial P | multifactorial | High | Normal | Normal | Negative | Negative | Negative | Negative | Negative | 39.0 | 3 | 24 | Survival |
| **14** | DT | 25 | Lobular P | fungal | High | Normal | High | Positive | Positive | Positive | Negative | Negative | 38.5 | 3 | 42 | Survival |

PN: patient number; SP: stages of pneumonia; DT: during induction therapy; AT: after induction therapy; MT: mean time from given first chemotherapy drug to pneumonia diagnosis (days); Type 1: type of imaging; Type 2: type of pathogen; Lobar P: lobar pneumonia, also known as alveolar pneumonia; Lobular P: lobular pneumonia, also known as bronchial pneumonia; Interstitial P: Interstitial pneumonia, inflammation dominated by lung interstitial; G-T: 1-3-β-D-glucan test (G Test); GM-T: galactomannan test (GM Test); AA: aspergillus antibody; CA: candida antibody; MPA: mycoplasma pneumoniae antibody; T: the highest temperature of the child during pneumonia (℃); DF; duration of fever (days); DA: duration of agranulocytosis (days)
